# Eltrombopag plus cyclosporine in refractory immune thrombocytopenia: a single-center study

**DOI:** 10.1016/j.rpth.2023.100279

**Published:** 2023-06-14

**Authors:** Yilei Hong, Yingying Shen, Qi Liu, Jingjie Dong, Jingjing Xiang, Yiping Shen, Liqiang Wu, Yuhong Zhou, Baodong Ye, Dijiong Wu

**Affiliations:** 1 The First School of Clinical Medicine, Zhejiang Chinese Medical University, Hangzhou, Zhejiang, China; 2 Department of Hematology, The First Affiliated Hospital of Zhejiang Chinese Medical University, Hangzhou, Zhejiang, China

**Keywords:** cyclosporine, efficacy, eltrombopag, thrombocytopenia, thrombopoietin receptor agonist

## Abstract

**Background:**

With the development of thrombopoietin receptor agonists, the prognosis of immune thrombocytopenia (ITP) in patients in whom there was a poor response to first-line treatment has greatly improved. However, there are still some patients who are refractory to eltrombopag.

**Objectives:**

To explore the efficacy and safety of eltrombopag combined with low-dose cyclosporine in the management of patients with refractory ITP.

**Methods:**

A total of 21 participants with ITP who failed to respond to multiple lines of therapy (including a daily dose of 75 mg of eltrombopag for at least 30 days) treated at The First Affiliated Hospital of Zhejiang Chinese Medical University between January 2018 and August 2022 were included. All enrolled patients subsequently received 50 mg of eltrombopag daily and low-dose cyclosporine (3 mg/kg/d, with an initial target concentration of 75-120 ng/mL). The efficacy and safety of the combined therapies were evaluated.

**Results:**

A total of 76.2% (16/21) of the patients responded to the combination of cyclosporine and eltrombopag, with a median time to response of 14.5 (range, 5-37) days. A complete response (platelet count ≥ 100 × 10^9^/L) was observed in 81.3% (13/16) of the patients, among whom 1 patient experienced relapse due to self-reduction in eltrombopag. During a median follow-up of 180 days, there were no relapses, and 70% (7/10) of the patients successfully stopped or decreased concomitant ITP medications. One patient had both a catheter-related deep vein thrombosis and a venous cerebral thrombotic event later; no other severe drug-related adverse events were observed.

**Conclusion:**

Combining low-dose cyclosporine with eltrombopag may be an effective alternative for multirefractory ITP that is nonresponsive to eltrombopag alone.

## Essentials

• The management of refractory immune thrombocytopenia (ITP) represents a substantial challenge.
• A combination of cyclosporine and eltrombopag induced a response in three-fourth of patients with refractory ITP.
• A combination of cyclosporine and eltrombopag can result in sustained remission for refractory ITP.
• Combination therapy was effective and well tolerated in most patients.

## 1 Introduction

Immune thrombocytopenia (ITP) is an autoimmune disease characterized by decreased platelet count and hemorrhagic tendency. Currently, the recommended first-line treatments for ITP include glucocorticoids, intravenous immunoglobulin, or anti-D immunoglobulin; however, only 60% to 80% of patients are responsive to these treatments [1]. Furthermore, about 40% to 60% of patients show relapse during glucocorticoid reduction or withdrawal and require a second-line treatment [2], which may include a thrombopoietin receptor agonist (TPO-RA: eltrombopag, herombopag, avatrombopag, and romiplostim), rituximab, and/or splenectomy. Moreover, second-line treatment in China also includes recombinant human thrombopoietin (rhTPO), a glycosylated full-length peptide thrombopoietin (3SBIO), which can achieve a response rate of 63.2%, with a median time to response of 7.5 days [3]. However, as it is unavailable in North America and most other Western countries, international guidelines recommend TPO-RA as a second-line treatment [[4], [5], [6]].

Eltrombopag is an orally absorbable, nonpeptide, small-molecule TPO-RA that provides a sustained response in 70% to 80% of patients receiving long-term treatment for chronic ITP [7] and a sustained remission rate of 30% after drug withdrawal [8]. However, the prognostic indicators for patients who fail to respond to initial treatment or experience relapse during treatment with eltrombopag are yet to be confirmed. As there is still no consensus on how to manage eltrombopag-resistant ITP, other TPO-RAs, fostamatinib [9] (syk inhibitor), efgartigimod [10] (FcRn antagonist), and other immune suppressive therapies, alone or in combination, may be considered.

According to Chinese guidelines on the diagnosis and management of adult ITP, immunosuppressive therapy (IST), such as cyclosporine A and azathioprine, and other regimens, including danazol and decitabine, are recommended for patients who fail to respond to second-line treatment [4]. However, the efficacy of mono-IST therapy has a prolonged onset time (6-8 weeks for cyclosporine A, with an overall response rate [ORR] of 62% [11]; 7 weeks for azathioprine, with an ORR of 73.33% [12]) and high incidence of adverse effects. Furthermore, only a few studies have reported the combined effect of eltrombopag with IST for refractory ITP. The total response to eltrombopag combined with azathioprine for patients with refractory ITP was significantly higher than that to azathioprine alone (90.32% vs 73.33%, respectively); however, the incidence of side effects was also significantly increased [[12], [13], [14]], particularly related to hepatotoxicity (∼25%). Thus far, only a limited number of studies have focused on improving the efficacy of eltrombopag-resistant ITP, achieving a response of about 40% to 50% [9,10]. Consequently, a combination treatment with a safe and rapid response should be explored, especially for eltrombopag-resistant ITP. Herein, we explored the efficacy and safety of eltrombopag combined with low-dose cyclosporine A in the management of patients with ITP who failed to respond to eltrombopag alone.

## 2 Methods

### 2.1 Study design

Patients with ITP who failed to respond to multiple treatments, including previous treatment with eltrombopag (at least 75 mg every day for 30 days), were recruited at The First Affiliated Hospital of Zhejiang Chinese Medical University (Hangzhou, China) between January 2018 and August 2022. All patients continued with eltrombopag at a dose of 50 mg daily and were orally administered low-dose cyclosporine A. Blood counts were monitored every 3 to 4 days until the platelet count reached 50 × 10^9^/L (during hospitalization) and monitored at least once a week after discharge throughout the study period. Safety was assessed by monitoring for treatment-emergent adverse events and serious adverse events throughout the study period.

The study was conducted in accordance with the Declaration of Helsinki. Written or oral informed consent was obtained from all patients. The present study was registered at chictr.org.cn as #ChiCTR2200062120.

### 2.2 Patient enrollment

The inclusion criteria for patients in keeping with the Consensus of Chinese Experts on Diagnosis and Treatment of Adult Primary ITP (version 2020) [4] were as follows: age ≥ 18 years; platelet count < 30 × 10^9^/L with or without bleeding symptoms before enrollment; failure to respond to multiple lines of therapy (the drugs were ineffective), patients were dependent on first-line glucocorticoid therapy, no response to previous rhTPO (at least 15,000 U every day for 14 days), and failed to respond to prior eltrombopag treatment (at least 75 mg every day for 30 days). The exclusion criteria were as follows: severe cardiopulmonary dysfunction, hepatic and renal insufficiency, immunodeficiency, pregnant or lactating women, and a history of thrombosis or other autoimmune diseases. In addition, bone marrow aspiration and biopsy were performed before eltrombopag administration; therefore, secondary thrombocytopenia (including myelodysplastic syndrome, aplastic anemia, and thrombocytopenia) was excluded.

### 2.3 Treatment

The participants received eltrombopag at an initial dose of 50 mg once daily and were orally administered cyclosporine A at a dosage of 3 mg/kg/d (given twice daily) to maintain the trough serum concentration at 75 to 120 ng/mL. Etrombopag and cyclosporine A were titrated according to the following protocol based on individual response. The overall goal of the dose modifications was to maintain the platelet count between ≥50 × 10^9^/L and ≤150 × 10^9^/L. Concurrent use of supportive care with platelet transfusion and rescue therapy for severe thrombocytopenia was considered if the platelet count was <10 × 10^9^/L or <20 × 10^9^/L with a bleeding symptom or risk factors [15].

### 2.4 Dose titration protocol

**Figure 1:**
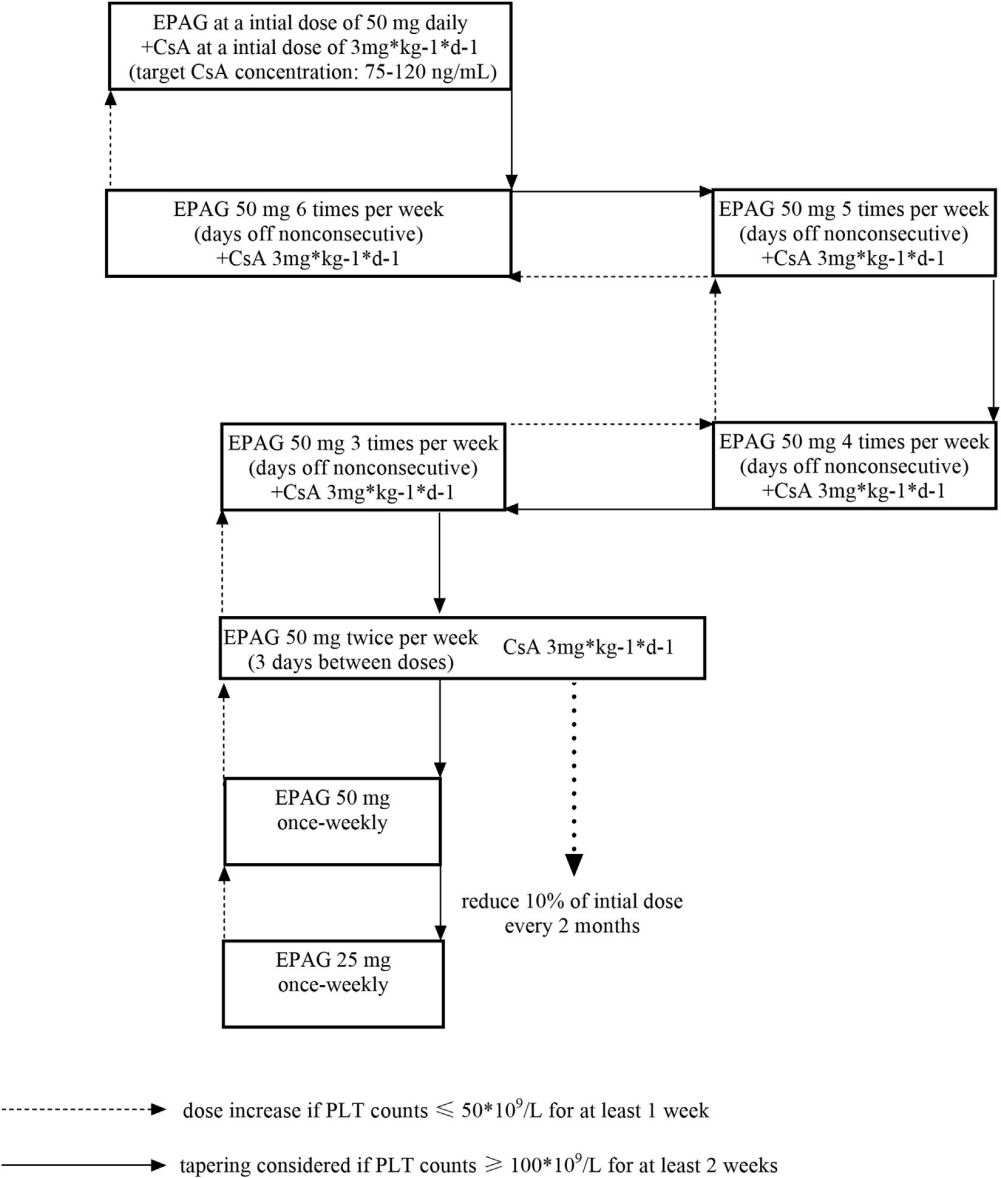
Dose titration protocol of the combined treatment. The dosage and frequency were adjusted according to individual response according to the titration protocol, as presented. The dose titration of EPAG was performed according to the following criterion for effective cases: (1) tapering considered if the platelet (PLT) count reached 100 × 10^9^/L for at least 2 weeks, (2) tapered immediately if the PLT count reached 200 × 10^9^/L-400×10^9^/L, and (3) restarted at the former dosage if the PLT decreased to 50 × 10^9^/L. The dose of cyclosporine began to decrease gradually when EPAG was administrated at 50 mg twice a week. CsA, cyclosporine; EPAG, eltrombopag; PLT, platelet.

Patients who did not respond to the combination treatment within the 16-week core administration period and those who were unwilling to continue treatment withdrew from the study. For cases in which the combination therapy was effective, the dose was adjusted according to the following scheme [16]: first, decreasing the dose of eltrombopag was considered. If the platelet count reached 100 × 10^9^/L and was maintained at this level for at least 2 weeks, a gradual reduction in the dosage was considered (the administration frequency was decreased by 1 day every 2 weeks if the platelet count was ≥50 × 10^9^/L without the stopping day; if the platelet count decreased to <50 × 10^9^/L, the dose was increased). If the platelet count reached 200 × 10^9^/L to 400 × 10^9^/L, eltrombopag administration was immediately ceased, and the patient was closely monitored. Administration of the drug was restarted at the former dosage if the platelet count decreased to 50 × 10^9^/L. All drug dose reductions first addressed the frequency and then the particular drug dosage. In this period, the dose of cyclosporine A was titrated to achieve the goal of 75 to 120 ng/mL. Second, the gradual decrease in cyclosporine A dose began when eltrombopag was administered at 50 mg twice per week; the dosage was adjusted every 2 months, with a 10% decline each time (Figure 1). During the reduction in cyclosporine A, the trough serum concentration was not required to maintain the stated goal.

### 2.5 Study assessment

The platelet counts were monitored accordingly, and the results were recorded and analyzed at weeks 1, 2, 3, 4, 6, 8, 14, and 16 after treatment. Response to treatment was defined as a platelet count of ≥30 × 10^9^/L and at least 2 times the baseline platelet count for at least 1 week, with no need for platelet transfusion. No response was defined as a platelet count of <30 × 10^9^/L after the initial combined treatment (with a maximum dosage of eltrombopag at 50 mg/d and cyclosporine A within the target concentration range) for >16 weeks, independent of platelet transfusion [4].

### 2.6 Study endpoints

The primary endpoint was the efficacy of eltrombopag combined with the cyclosporine A regimen at week 16 after treatment and the duration of a platelet count of ≥50 × 10^9^/L by monitoring changes in platelet counts. The secondary endpoints included the following: concomitant ITP drug use, bleeding symptoms, and treatment-emergent adverse events and serious adverse events over the course of the study period.

### 2.7 Response assessment

Complete blood count was performed at least twice a week during the first month of treatment. Complete response was defined as a platelet count of ≥100 × 10^9^/L without bleeding symptoms; partial response was defined as a platelet count of ≥30 × 10^9^/L and at least 2 folds of the baseline platelet count, without bleeding symptoms; and no response was defined as a platelet count of <30 × 10^9^/L, increase in platelet count by less than twice the baseline value, or occurrence of bleeding symptoms. Relapse was diagnosed when the patients failed to meet the response criteria. To ensure accuracy, 2 lowest platelet counts recorded at least 1 day apart were used [4].

### 2.8 Bleeding assessment

**Figure 2:**
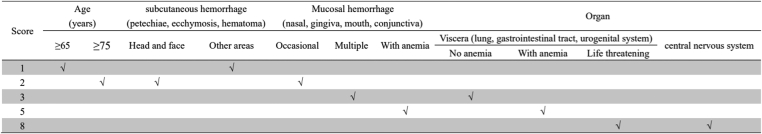
Bleeding score system (Chinese guidelines on the diagnosis and management of adult primary immune thrombocytopenia). The most severe bleeding events that occurred in the skin, mucous, and organ during the course of the disease were recorded; age was taken into account as a risk factor; total bleeding scores were calculated by summing the scores on the 4 components.

Bleeding was assessed at baseline and at week 16 after therapy initiation. Bleeding grading was performed according to World Health Organization criteria, wherein grade 1 indicates petechiae, grade 2 indicates mild blood loss, grade 3 indicates gross blood loss, and grade 4 indicates debilitating blood loss [17]. The bleeding score of all patients was assessed according to the bleeding assessment table in Chinese guidelines on the diagnosis and management of adult primary ITP (version 2020) [4], which include the assessment of bleeding symptoms in the skin, mucosa, and deep organs and recording the most severe bleeding during the course of the disease; taking age into account as a risk factor; and calculation of total bleeding scores by summing the scores for each item (Figure 2).

### 2.9 Safety observation

Blood biochemistry and cyclosporine A concentration were detected at least twice a week during the first month of treatment, and blood pressure was measured once every morning and evening. Subsequently, the laboratory examination of blood was monitored every 2 weeks for patients who were followed up for at least 3 months. Additionally, adverse effects, such as gingival hyperplasia, gastrointestinal reactions, hirsutism, tremors, and pulmonary infections, were monitored at the baseline and at 16 weeks after treatment. Doppler ultrasound and electrocardiography of the spleen, liver, and lower-extremity deep bilateral vein were performed to evaluate the safety of the treatment.

### 2.10 Statistical analysis

SPSS, version 22, was used for all statistical analyses. Numerical data were reported using descriptive statistics, including frequencies, medians, and ranges. The Student’s *t*-test was used to compare the differences in the bleeding scores before and after treatment. Categorical data were presented as numbers and percentages. *P* <.05 was considered statistically significant.

## 3 Results

### 3.1 Patient characteristics

A total of 21 patients with ITP, including 6 men and 15 women with a median age of 48 (range, 18-79) years, who failed to respond to multiple lines of treatment were enrolled in this study. Their median duration of the disease was 12 (range, 3-360) months, and the median platelet count before treatment was 8 × 10^9^/L (range, 2 × 10^9^/L-28 × 10^9^/L) and was associated with varying degrees of bleeding and a median bleeding score of 3 (range, 1-9). The median time of continuous treatment with eltrombopag before study entry was 41 (range, 30-100) days, and that of continuous treatment with rhTPO was 73.5 (range, 15-96) days. Patient (PT) 1, PT2, PT14, PT15, PT17, and PT21 responded to initial treatment with eltrombopag; PT2, PT14, and PT15 discontinued eltrombopag on their own initiative due to economic difficulties, while PT1 and PT17 suspended the use of eltrombopag due to hepatotoxicity. As a result, their platelet counts rapidly dropped to initial levels, and some patients had various bleeding symptoms. All patients reinitiated eltrombopag before enrollment; however, no response was observed. Additionally, patients who previously failed to respond to splenectomy and rituximab regimens were enrolled in this study (Table, Supplementary Table).

**Table:** General data of the enrolled 21 patients.

PT	Previous treatment regimens	Concomitant medication/course, dosage	Bleeding score before treatment	Initial PLT (× 10^9^/L)	Response time (d)	Bleeding score after 16 wk	Follow-up time (d)	Duration of PLT count of ≥50 × 10^9^/L (d)
SR1	G^1^I^1^T^1^E^1^S	/	4	8	17	1	550	530
SD2	G^1^IT^1^E^1^	/	4	6	37	1	557	511
SR3	GITES	T/60 d, 15,000 U/d	3	14	22	3	553	400
SD4	G^1^ITE	G/33 d, 12 mg/d	6	9	/	6	232	0
SR5	GITER	G/37 d, 20 mg/d D/35 d, 200 mg/d	3	6	16	0	100	81
SD6	GITER	D/60 d, 200 mg/d	3	5	12	0	1310	1283
SR7	GT^1^E	/	3	10	5	0	140	132
SR8	GITER	/	5	3	/	3	195	0
SR9	GT^1^E	/	3	14	/	3	57	0
SR10	G^1^I^1^T^1^E	G/55 d, 20 mg/d	1	9	11	0	367	336
SD11	G^1^I^1^T^1^E	G/40 d, 20 mg/d	3	8	7	0	243	64
SR12	G^1^ITER	G/35 d, 5 mg/d	9	3	14	0	535	519
SD13	GIT^1^ER	/	9	2	15	0	501	466
SR14	GTE^1^	T/115 d, 15,000 U/d	4	19	35	3	137	18
SR15	G^1^I^1^TE^1^	/	3	16	23	0	70	47
SD16	G^1^I^1^TE	/	5	5	13	1	65	52
SR17	GIT^1^E^1^S	/	2	28	8	2	61	40
SR18	G^1^ITE	G/28 d, 20 mg/d	3	9	/	3	60	0
SR19	G^1^T^1^E	G/32 d, 12 mg/d	1	7	24	0	57	32
SD20	GITE	/	2	4	/	1	180	0
SD21	GT^1^E^1^	/	3	9	11	0	33	22

1, initial treatment is effective and the subsequent treatment is ineffective; D, danazol; E, eltrombopag olamine tablets (revolade); G, glucocorticoid; I, human intravenous immunoglobulin; R, rituximab; S, splenectomy; SD, steroid dependence; SR, steroid resistance; T, recombinant human thrombopoietin.

### 3.2 Concomitant medications

All enrolled patients were previously refractory to ITP monotherapy. A certain number of patients continued to receive concomitant ITP treatment before enrollment, as listed in the Table, which was then gradually decreased according to the platelet count response. The dosage or frequency of administration was gradually reduced if the platelet count was ≥50 × 10^9^/L after study entry and stopped when the platelet count reached 100 × 10^9^/L for at least 2 weeks. During the follow-up period, 47.6% (10/21) of the patients received concomitant medication at the beginning of treatment. PT4, PT10, PT11, PT12, PT18, and PT19 received concomitant glucocorticoids. The medication coadministered in PT3 and PT14 was rhTPO, while danazol was used in PT6. PT5 was the only one who received 2 concomitant medications, ie, glucocorticoid and danazol. PT3, PT4, PT5, PT10, PT11, and PT12, accounting for 60% (6/10) of the patients, gradually decreased and finally stopped concomitant medication, with a median duration time of 30 (range, 11-100) days. The low-dose glucocorticoid was successfully decreased in 1 case (PT19), but withdrawal failed at the end of follow-up.

### 3.3 Efficacy

**Figure 3:**
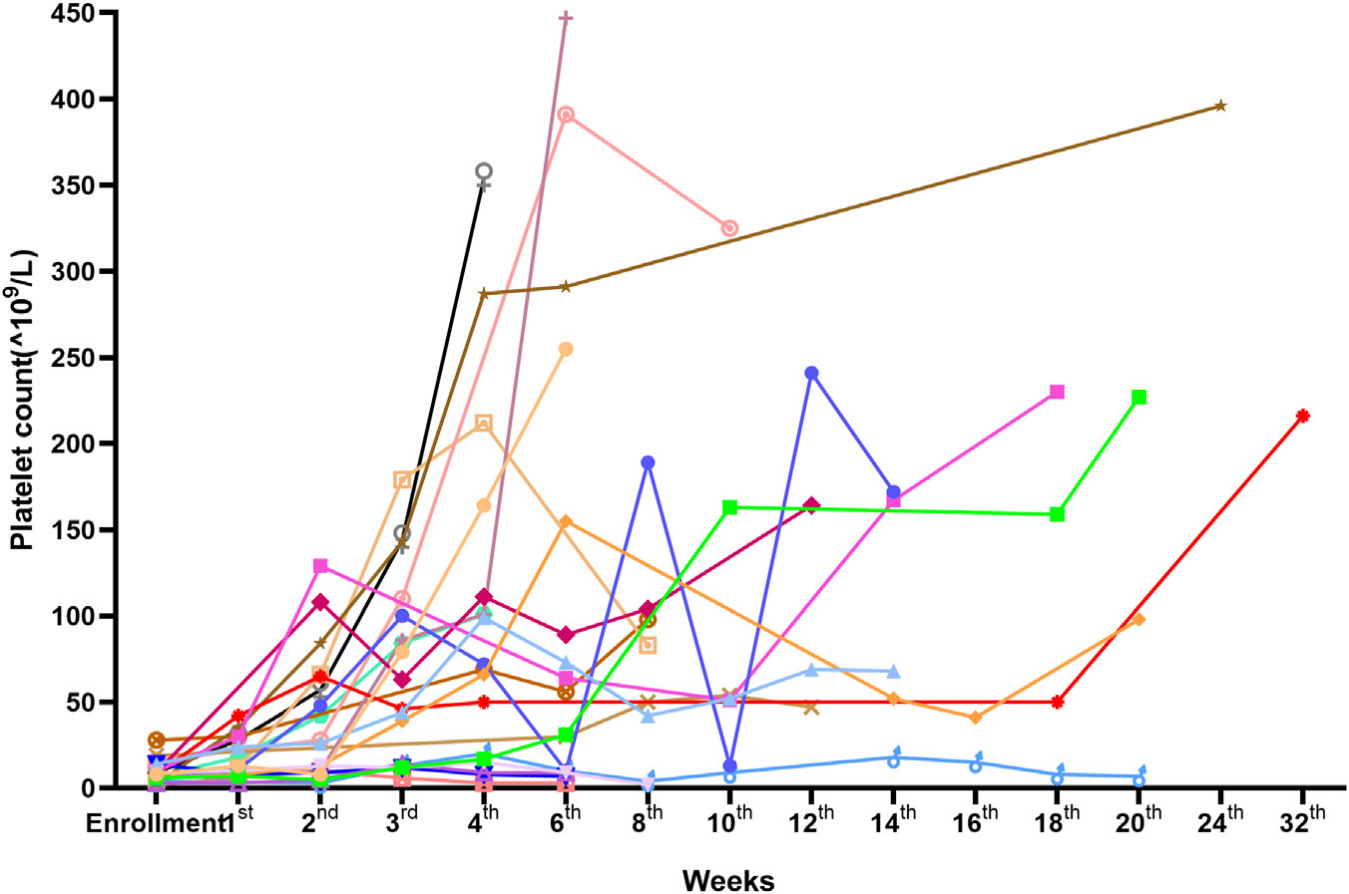
Individual changes in platelet count during combined treatment with EPAG and cyclosporine. In total, 76.2% (16/21) of the patients responded to combined treatment with EPAG and cyclosporine, and the median response time was 14.5 (range, 5-37) days. For the 16 patients who responded, 12 achieved continuously complete response during the follow-up period, 1 patient experienced relapse from complete response due to self-reduction in EPAG and regained therapeutic response after EPAG was restarted. EPAG, eltrombopag.

A positive response to eltrombopag combined with cyclosporine A treatment in a median duration of 14.5 (range, 5-37) days was observed in 76.2% (16/21) of the patients. Of the 16 responders, 13 exhibited complete response, among whom 12 achieved sustained response during the follow-up period. PT5 showed a decreased platelet count due to self-reduction in the eltrombopag dosage, which subsequently returned to normal. During a median follow-up of 180 days, no relapse was observed in these 16 patients. The median duration of sustained platelet response was 106.5 (range, 18-563) days (Figure 3). To consider whether steroid resistance was an adverse predictive factor for response to the combination of eltrombopag and cyclosporine A, we compared the responses based on those with previous steroid resistance or steroid dependence (Table) and found a response proportion of 76.9% (10/13) in the steroid resistance group compared with 75% (6/8) in the steroid dependence group; no significant differences were observed (*P* = .920).

### 3.4 Bleeding symptom

**Figure 4:**
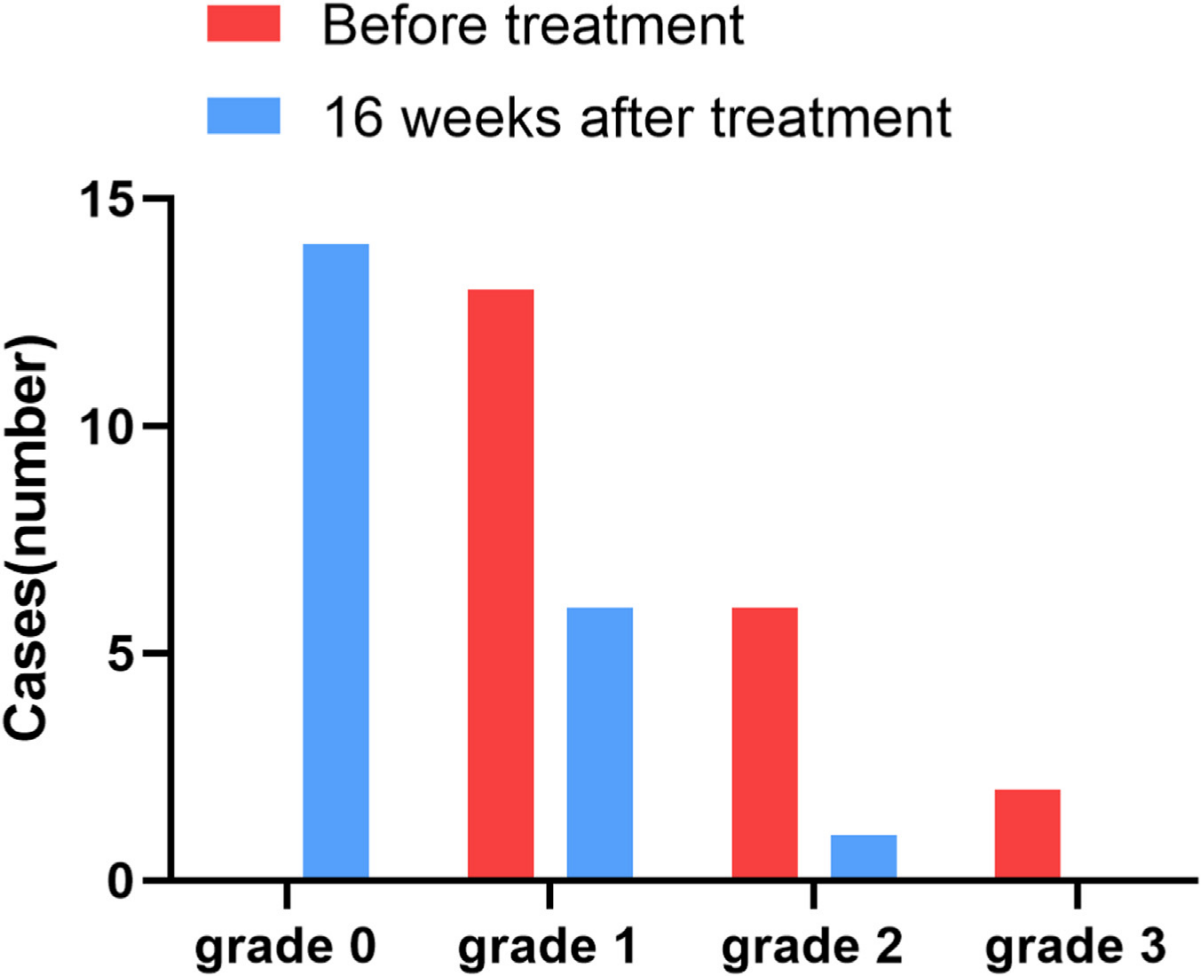
Distribution of bleeding grade before and after combined treatment with EPAG and cyclosporine. Bleeding symptoms occurred in all patients (100%) at baseline and decreased to 33.3% (7/21) after 16 weeks of treatment. Grade 1 bleeding mostly manifested with petechiae; grade 2 bleeding mainly manifested as oral bleeding, epistaxis, and gastrointestinal bleeding; in 2 patients, grade 3 bleeding was related to gastrointestinal tract and urogenital hemorrhage; no grade 4 bleeding was observed.

All patients had bleeding symptoms before treatment, which decreased to 33.3% (7/21) at the end of follow-up. The median bleeding score significantly decreased from 3 (range, 1-9) at the baseline to 1 (range, 0-6) after a follow-up of 16 weeks (*P* = .000238). Among these patients with bleeding symptoms at the end of follow-up, 85.7% (6/7) presented with grade 1 bleeding (petechiae) and only 1 patient had grade 2 bleeding (oral bleeding) (Figure 4).

### 3.5 Adverse effects

The incidence of adverse events was 19% (4/21). Three cases presented with grade 1 to 2 adverse events, which included gingival hyperplasia and mild gastrointestinal reactions, which were alleviated after supportive treatment of symptoms. PT6 experienced a grade 3 adverse event, wherein right upper-extremity venous catheter–associated thrombosis developed 12 days after the eltrombopag and cyclosporine A combination treatment was started (with a platelet count of 100 × 10^9^/L). The patient immediately received low-molecular–weight heparin, and the central line was removed 7 days later. Due to the encouraging platelet count response and other provoking risk factor for the thrombotic event, the treatment was continued. After 14 days of anticoagulation therapy, Doppler ultrasound showed that the size of the thrombus decreased from 3.2 cm to 2.2 cm, and the anticoagulation therapy was stopped due to the decreased platelet count (∼18 × 10^9^/L), with close monitoring. However, 2.5 months later, the patient had a seizure, and the platelet count rose to 446 × 10^9^/L. Magnetic resonance imaging showed acute cerebral infarction of the right frontal and parietal lobes, while magnetic resonance venography confirmed partial venous thrombosis in the right parietal lobe. Neurologists and neurosurgeons speculated that the cerebral venous infarct was independent of the prior upper-extremity venous thrombosis and was unsuitable for thrombolysis or surgical embolectomy. According to multidisciplinary consultation opinions, aspirin (100 mg every day), levetiracetam (0.5 g twice a day), valproic acid (0.4 g every day), and midazolam (30 mg every day) were added, and the patient’s symptoms were relieved, with a stable platelet count of ∼80 × 10^9^/L. Treatment with 25 mg of eltrombopag every day and 100 mg of cyclosporine A twice a day was continued, and the thrombus had resolved at follow-up. No other obvious adverse effects (including gingival hyperplasia and gastrointestinal side effects) were detected in the remaining patients, and their liver function parameters were maintained within normal limits.

## 4 Discussion

This interventional study aimed to assess the efficacy and safety of combination treatment with eltrombopag and cyclosporine A for primary ITP patients who refractory or relapse from eltrombopag monotherapy with or without other concomitant medications. We found that 61.9% (13/21) of the patients responded completely and 14.3% (3/21) responded partially to combination therapy, achieving an ORR of 76.2%. No relapse was observed after a median follow-up of 180 days.

In recent years, the efficacy and safety of eltrombopag as a small, second-generation, nonpeptide TPO-RA used for second-line treatment has been well recognized. However, the sustained response rate after drug withdrawal is low, and long-term medication is required to maintain the response. In addition, some patients with ITP cannot achieve long-term remission from eltrombopag. Qu et al. [18] showed that monotherapy with TPO-RAs increases the level of transforming growth factor-β1 but has no significant effect on improving the immune imbalance of Th1 cytokines in patients with ITP, thus providing a theoretical basis for the feasibility of multidrug combination therapy with eltrombopag. Conversely, cyclosporine A, a commonly used immunosuppressant, inhibits the expression of interleukin-2 in cytotoxic T lymphocytes and helper T lymphocytes (Th) and inhibits Th cells from producing and releasing interleukin-2. Therefore, the complementary mechanisms of eltrombopag and cyclosporine A may be exploited to increase the platelet count. Furthermore, it has been reported that the optimal trough concentration of monotherapy cyclosporine A in patients with ITP is >120 ng/mL to maintain the response, which may increase the risk of adverse effects [19]. In order to minimize this risk, we maintained the concentration of cyclosporine A between 75 and 120 ng/mL in combination therapy.

The same findings were observed in patients who were previously steroid resistant or steroid dependent. In addition, during the follow-up period, the eltrombopag dosage was successfully decreased in 9 patients, and the platelet count continued to show a favorable response. All these results suggest that the combination therapy of low-dose cyclosporine A and eltrombopag should be considered as an alternative therapeutic modality for ITP that has previously failed to respond to multiple lines of therapy. However, this was a single-arm, single-center study with a limited sample size, and some patients were followed up for a short period of time. Importantly, the participants did not previously receive cyclosporine A monotherapy, which made it difficult to evaluate and compare their response to single-agent cyclosporine A management. Thus, future randomized trials are needed to further evaluate this promising drug therapy combination.

## 5 Conclusion

The combination therapy of eltrombopag and cyclosporine A may exploit complementary drug mechanisms in refractory ITP. Future large, randomized trials are needed to evaluate this therapeutic modality for refractory ITP.
