# Supplementary material for: Eltrombopag plus cyclosporine in refractory immune thrombocytopenia: a single-center study
Source: Res Pract Thromb Haemost. 2023 Jun 14;7(5):100279. doi: 10.1016/j.rpth.2023.100279 (PMC10339056; doi:10.1016/j.rpth.2023.100279)
Supplement: Supplementary Table [file mmc1.docx]

**Supplementary Table. Demographic information of enrolled patients**

| PT | Race | Gender  (F/M) | Age  (years) | Course of  ITP (Y/M) | Previous treatment regimens(numbers) | Concomitant medication(numbers) |
| --- | --- | --- | --- | --- | --- | --- |
| 1 | Asian | F | 65 | 1Y | 5 | 0 |
| 2 | Asian | F | 69 | 3Y | 4 | 0 |
| 3 | Asian | M | 56 | 20Y | 5 | 1 |
| 4 | Asian | F | 79 | 8Y | 4 | 1 |
| 5 | Asian | M | 44 | 4M | 5 | 2 |
| 6 | Asian | M | 22 | 7M | 5 | 1 |
| 7 | Asian | M | 48 | 3M | 3 | 0 |
| 8 | Asian | F | 63 | 30Y | 5 | 0 |
| 9 | Asian | F | 49 | 7M | 3 | 0 |
| 10 | Asian | F | 60 | 4M | 4 | 1 |
| 11 | Asian | F | 46 | 10M | 4 | 1 |
| 12 | Asian | F | 32 | 5Y | 5 | 1 |
| 13 | Asian | F | 40 | 5M | 5 | 0 |
| 14 | Asian | F | 58 | 7Y | 3 | 1 |
| 15 | Asian | F | 25 | 8Y | 4 | 0 |
| 16 | Asian | F | 65 | 4M | 4 | 0 |
| 17 | Asian | M | 74 | 1Y | 5 | 0 |
| 18 | Asian | M | 48 | 8Y | 4 | 1 |
| 19 | Asian | F | 18 | 2Y | 3 | 1 |
| 20 | Asian | F | 36 | 10Y | 4 | 0 |
| 21 | Asian | F | 29 | 1Y | 3 | 0 |

Note: F: female; M: male; Y: year(s); M: month(s);
